# Association Between Early Mobilization and Postoperative Pneumonia Following Robot-assisted Minimally Invasive Esophagectomy in Patients with Thoracic Esophageal Squamous Cell Carcinoma

**DOI:** 10.1298/ptr.E10293

**Published:** 2024-09-20

**Authors:** Yasuaki NOZAWA, Kazuhiro HARADA, Kazuhiro NOMA, Yoshimi KATAYAMA, Masanori HAMADA, Toshifumi OZAKI

**Affiliations:** 1 Division of Physical Medicine and Rehabilitation, Okayama University Hospital, Japan; 2 Graduate School of Health Science Studies, Kibi International University, Japan; 3 Department of Gastroenterological Surgery, Graduate School of Medicine, Dentistry and Pharmaceutical Sciences, Okayama University, Japan

**Keywords:** Early mobilization, Postoperative pneumonia, Orthostatic intolerance, Thoracic esophageal squamous cell carcinoma, Robot-assisted minimally invasive esophagectomy

## Abstract

Objective: The objective of this study was to confirm that early mobilization (EM) could reduce pneumonia in patients undergoing robot-assisted minimally invasive esophagectomy (RAMIE) for thoracic esophageal squamous cell carcinoma (TESCC). Methods: Postoperative pneumonia was defined as physician-diagnosed pneumonia using the Esophagectomy Complications Consensus Group definition of pneumonia with a Clavien–Dindo classification grade II–V on postoperative day (POD) 3–5. EM was defined as achieving an ICU Mobility Scale (IMS) ≥7 by POD 2. Patients were divided into EM (n = 36) and non-EM (n = 35) groups. Barriers to EM included pain, orthostatic intolerance (OI), and orthostatic hypotension. Results: The overall incidence of postoperative pneumonia was 12.7%, with a significant difference between the EM (2.8%) and non-EM (22.9%) groups (P = 0.014). The odds ratio was 0.098 in the EM group compared to the non-EM group. A significant difference was found between the two groups in terms of the barriers to EM at POD 2 only for OI, with a higher incidence in the non-EM group. Multivariate logistic regression analysis showed that patients with OI were more likely to be unable to achieve EM than those without OI (odds ratio, 7.030; P = 0.006). Conclusion: EM within POD 2 may reduce the incidence of postoperative pneumonia in patients undergoing RAMIE for TESCC. Furthermore, it was suggested that OI can have a negative impact on the EM after RAMIE.

## Introduction

The incidence of pneumonia following esophagectomy has been reported to range from 13.9% to 21.0%^1^^–^^4)^ and is associated with a decrease in overall survival^5–7)^. Tanaka et al.^8)^ reported that acute-phase pneumonia within 7 days after esophagectomy for esophageal squamous cell carcinoma affected overall survival and recurrence; however, subacute-phase pneumonia after 8 days had no effect on prognosis. Therefore, the prevention of acute-phase pneumonia in the short term following esophagectomy may improve long-term prognosis, including overall survival and recurrence.

Robot-assisted minimally invasive esophagectomy (RAMIE) is a newly introduced approach for minimally invasive esophagectomy. A previous systematic review and meta-analysis^9^^–^^11)^ that compared RAMIE with conventional minimally invasive esophagectomy reported a lower incidence of postoperative pneumonia.

Immobilization is known to cause postoperative pneumonia, while secretion retention owing to immobilization has been linked specifically with bacterial pneumonia^12)^. Guidelines for perioperative care following esophagectomy^13)^ state that early mobilization (EM) not only helps to preserve muscle function and prevent complications associated with bed rest but also empowers patients to take an active role in their recovery from surgery, with a moderate level of evidence and a strong recommendation grade. Studies investigating the association between EM and postoperative complications (including pulmonary complications) have shown that EM is associated with a lower incidence of postoperative complications^14–18)^, whereas other studies have shown no association^19^^,^^20)^. Existing studies on the subject^14^^–^^20)^ also carry several limitations: (1) The definitions of EM and postoperative pulmonary complications varied among the studies. (2) They did not consider the temporal relationship between EM and postoperative pulmonary complications. (3) These studies were not limited to patients with esophageal cancer because of the wide variety of diseases and surgical procedures.

The clarification of the association between EM and a lower incidence of postoperative pneumonia would be of clinical significance because such knowledge could improve overall survival and recurrence rates. In addition, defining EM and postoperative pneumonia using an assessment tool would be reproducible and would have research significance because it would allow follow-up studies on the association between physical activity and postoperative pneumonia in the intensive care unit (ICU).

The objective of this study was to confirm that EM could reduce pneumonia in patients undergoing RAMIE for thoracic esophageal squamous cell carcinoma (TESCC).

## Methods

### Study design

This was a single retrospective cohort study conducted at the Okayama University Hospital.

### Participants

Patients who underwent RAMIE from October 1, 2018 to September 30, 2022 were enrolled through convenience sampling.

The inclusion criteria were as follows: (1) patients who received perioperative care at our hospital; (2) male sex; (3) main location of the lesion in the thoracic esophagus; (4) histologic type: SCC; (5) able to be weaned from the ventilator by postoperative day (POD) 1; (6) underwent patient-controlled analgesia (PCA) for postoperative pain management; (7) enteral nutrition was used for postoperative nutrition management; and (8) patients who met the criteria for EM (including out-of-bed mobilization) at our hospital and were able to start on POD 1.

Patients who met the following criteria were excluded because they may affect EM, postoperative pneumonia, and test results: (1) female sex; (2) cognitive impairment; (3) preoperative motor dysfunction; (4) two-stage esophagectomy, salvage surgery, concurrent surgery, etc.; (5) early postoperative limitation of mobilization; (6) delirium within POD 2; and (7) pneumonia within POD 2.

### Definition of postoperative pneumonia

The Esophagectomy Complications Consensus Group (ECCG)^21)^ defines pneumonia as “new lung infiltrates plus clinical evidence that the infiltrate is of an infectious origin, which includes the new onset of fever, purulent sputum, leukocytosis, and decline in oxygenation.” The Clavien–Dindo classification^22)^ is primarily used for adverse events related to early postoperative complications.

Patients diagnosed with postoperative pneumonia on POD 2 were excluded because of the temporal relationship between EM and postoperative pneumonia. The average ICU stay following esophagectomy at our hospital was approximately 5 days. Therefore, postoperative pneumonia in this study was defined as pneumonia diagnosed by a physician using the ECCG definition of pneumonia with a Clavien–Dindo classification grade II–V on POD 3–5.

### Definition and classification of EM

The Japanese Society of Intensive Care Medicine defines early rehabilitation as a “series of interventions to maintain, improve, or regain movement, respiratory function, etc., initiated within 48 hrs of new onset, surgery, or acute disease”^23)^.

In this study, EM was assessed using the ICU Mobility Scale (IMS)^24)^. Out-of-bed mobilization in this study was defined as sitting over the edge of the bed to walking^25^^,^^26)^. In addition, EM was defined as achieving ≥IMS7 (able to walk away from the bed/chair by at least 5 m [5 yards] assisted by two or more people) by POD 2. Those who were able to achieve EM by POD 2 were classified as the EM group, and those who had difficulty were classified as the non-EM group.

### Definition of barriers to EM

Pain was defined as the maximum pain experienced during EM assessed using a Numerical Rating Scale (NRS). Orthostatic intolerance (OI)^27)^ was defined as the occurrence of any of the following symptoms upon mobilization to sitting in bed, sitting over the edge of the bed, or standing: lightheadedness, headache, fatigue, nausea, sweating, or visual disturbances. Orthostatic hypotension (OH)^28)^ was defined as a decrease in systolic blood pressure of at least 20 mmHg or diastolic blood pressure of 10 mmHg upon mobilization to sitting in bed, sitting over the edge of the bed, or standing.

### Primary and secondary outcomes

The primary outcome of this study was the incidence of postoperative pneumonia. The secondary outcomes were the date of ventilator weaning, date of initiation of sitting over the edge of bed and standing, IMS on POD 1, barriers to EM (NRS, OI, and OH) on POD 2, delirium after POD 3, postoperative ICU stay, postoperative hospital stay, and 30- and 90-day mortality rates.

### Risk factors for postoperative pneumonia

The preoperative risk factors for postoperative pneumonia included age, body mass index, Brinkman index, albumin level, C-reactive protein level, neoadjuvant chemotherapy, neoadjuvant chemoradiotherapy, chronic obstructive pulmonary disease, diabetes mellitus, % vital capacity, percent of forced expiratory volume in 1 sec, gait speed, grip strength, and third lumbar skeletal muscle mass. Intraoperative risk factors included pathological stage, operative time, blood loss, and gastric conduit reconstruction (hand-assisted laparoscopic surgery or laparotomy). Postoperative factors were PCA (epidural or intravenous) and recurrent laryngeal nerve paralysis.

### Ethical considerations

This study was conducted with the approval of the Okayama University Ethics Review Committee (Lab 2203-047). All information was disclosed to the participants, with an opportunity to refuse the research (opt-out), and a document approved by the Okayama University Ethics Review Committee was posted on the website of the Division of Physical Medicine and Rehabilitation, Okayama University Hospital.

### Statistical analysis

This study did not perform a sample size calculation. However, we performed a post hoc power analysis based on the actual observed incidence of postoperative pneumonia and calculated it using G*Power software (version 3.1.9.7) (Heinrich Heine Universitat Dusseldorf, Dusseldorf, Germany).

The primary and secondary outcomes and risk factors for postoperative pneumonia were compared between the two groups. Differences were considered significant if the P-value was <0.05. Ratio scales were described as mean ± standard deviation or median (interquartile range) after confirming normality by the Shapiro–Wilk test and were compared by Student’s *t*-test or Mann–Whitney *U* test. Ordinal scales were described as medians (interquartile ranges) and compared using the Mann–Whitney *U* test. Nominal scales were described as the number of persons (%) and compared using Fisher’s exact test.

Multivariate logistic regression analysis was applied to determine the association between EM and barriers to EM. The dependent variable was the ability to perform ≥IMS7 on POD 2. The independent variables were the P-value <0.05 for the comparison between the two groups of barriers to EM (NRS, OI, and OH) at POD 2 and the P-value <0.1 for the comparison between the two groups of risk factors for postoperative pneumonia, to adjust for background factors, using forced entry methods. Statistical analyses were performed using EZR version 1.54 (Saitama Medical Center, Jichi Medical University, Saitama, Japan)^29)^.

## Results

### Patient characteristics

**Fig. 1.**
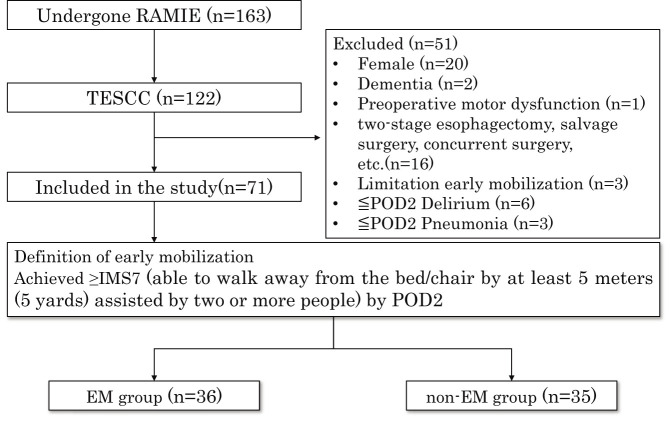
Subject of this study

From October 1, 2018 to September 30, 2022, 163 patients underwent RAMIE, of whom 122 were diagnosed with TESCC. Of these, 51 patients were excluded because they were female (n = 20), had dementia (n = 2), preoperative motor dysfunction (n = 1), surgical details (n = 16), limited EM (n = 3), delirium within POD 2 (n = 6), and pneumonia within POD 2 (n = 3). Finally, 71 patients were included in the study, of whom 36 were classified into the EM group and 35 into the non-EM group (Fig. 1).

IMS at POD 2 was 7.0 (7.0–7.0) in the EM group and 4.0 (3.0–4.0) in the non-EM group. The median start of ambulation was 2.0 (2.0–2.0) days in the EM group and 3.0 (3.0–4.0) days in the non-EM group.

### Risk factors for postoperative pneumonia

A comparison of risk factors for postoperative pneumonia between the two groups is shown in Table 1. There were no significant differences in any of the preoperative, intraoperative, or postoperative factors between the two groups.

**Table 1.** Comparison of risk factors for postoperative pneumonia between the two groups

Variable	Total (n = 71)	EM group (n = 36)	Non-EM group (n = 35)	P value
Age (years)	69.0 (62.0–73.0)	69.0 (63.5–72.0)	69.0 (62.0–75.0)	0.584
Body mass index (kg/m^2^)	22.7 ± 2.9	22.7 ± 3.0	22.6 ± 2.8	0.894
Brinkman index	750.0 (390.0–920.0)	570.0 (290.0–895.0)	800.0 (525.0–950.0)	0.269
Albumin (g/dl)	3.8 ± 0.4	3.8 ± 0.4	3.7 ± 0.4	0.058
C-reactive protein (mg/dl)	0.10 (0.05–0.18)	0.09 (0.05–0.14)	0.12 (0.06–0.23)	0.172
Neoadjuvant chemotherapy, n (%)	43 (60.6%)	21 (58.3%)	22 (62.9%)	0.809
Neoadjuvant chemoradiotherapy, n (%)	1 (1.4%)	0 (0.0%)	1 (2.9%)	0.493
Chronic obstructive pulmonary disease, n (%)	3 (4.2%)	2 (5.6%)	1 (2.9%)	1.000
Diabetes mellitus, n (%)	8 (11.3%)	3 (8.3%)	5 (14.3%)	0.478
% vital capacity (%)	103.4 (94.2–110.7)	107.5 (94.5–113.4)	100.5 (93.9–107.4)	0.077
Percent of forced expiratory volume in 1 sec (%)	76.50 (71.31–79.94)	77.10 (73.84–79.54)	75.50 (70.12–80.19)	0.508
Gait speed (m/s)	1.34 (1.21–1.51)	1.37 (1.25–1.61)	1.28 (1.12–1.42)	0.070
Grip strength (kg)	36.5 ± 6.1	37.9 ± 5.3	35.1 ± 6.7	0.065
Third lumbar skeletal muscle mass (cm^2^/m^2^)	45.2 ± 6.7	45.8 ± 6.5	44.5 ± 6.9	0.444
Pathological Stage 0/I/II/III/IV, n (%)	14/17/14/21/5 (19.7%/23.9%/19.7%/29.6%/7.0%)	7/10/8/9/2 (19.4%/27.8%/22.2%/25.0%/5.6%)	7/7/6/12/3 (20.0%/20.0%/17.1%/34.3%/8.6%)	0.849
Operative time (min)	591.0 (531.0–652.0)	582.5 (529.3–642.3)	597.0 (555.0–665.0)	0.384
Blood loss (ml)	145.0 (93.8–196.3)	120.0 (85.0–170.0)	150.0 (110.0–220.0)	0.051
Gastric conduit reconstruction (hand-assisted laparoscopic surgery: laparotomy), n (%)	64 (90.1%): 7 (9.9%)	35 (97.2%): 1 (2.8%)	29 (82.9): 6 (17.1%)	0.055
Patient-controlled analgesia (PCEA: IV-PCA), n (%)	62 (87.3%): 9 (12.7%)	29 (80.6%): 7 (19.4%)	33 (94.3%): 2 (5.7%)	0.151
Recurrent laryngeal nerve paralysis, n (%)	13 (18.3%)	5 (13.9%)	8 (22.9%)	0.372

This study had missing data. Gait speed: EM group (n = 1), non-EM group (n = 3); grip strength: EM group (n = 2), non-EM group (n = 2); third lumbar skeletal muscle mass: EM group (n = 1), non-EM group (n = 5); operative time: non-EM group (n = 2); and blood loss: EM group (n = 1), non-EM group (n = 2).

EM, early mobilization; PCEA, epidural patient-controlled analgesia; IV-PCA intravenous patient-controlled analgesia

### Primary outcome

Table 2 presents a comparison of the primary outcomes between the two groups. The overall incidence rate of postoperative pneumonia was 12.7% (n = 9/71). The incidence of postoperative pneumonia in the two groups was 2.8% (n = 1/36) in the EM group and 22.9% (n = 8/35) in the non-EM group, representing a significant difference between the two groups (P = 0.014). The odds ratio was 0.098 in the EM group compared to the non-EM group.

**Table 2.** Comparison of the primary outcome between the two groups

Variable	Total (n = 71)	EM group (n = 36)	Non-EM group (n = 35)	P value
Incidence of postoperative pneumonia, n (%)	9 (12.7%)	1 (2.8%)	8 (22.9%)	0.014

EM, early mobilization

### Post hoc power analysis for incidence of postoperative pneumonia

A post hoc power analysis for the incidence of postoperative pneumonia was calculated using an effect size of 1.218, a significance level of 5%, and a total sample size of 71. The results showed high statistical power (1.00). This shows that the sample size of 71 was adequate and that the study was sufficiently powered to detect an association between EM and the incidence of postoperative pneumonia.

### Secondary outcome

Table 3 presents a comparison of the secondary outcomes between the two groups. The EM group had a significantly earlier start date for sitting over the edge of the bed and standing, and a significantly higher IMS at POD 1. The ICU and postoperative hospital stays were significantly shorter in the EM group. None of the patients died within 90 days of surgery.

**Table 3.** Comparison of the secondary outcomes between the two groups

Variable	Total (n = 71)	EM group (n = 36)	Non-EM group (n = 35)	P value
Ventilator weaning (day)	1.0 (1.0–1.0)	1.0 (1.0–1.0)	1.0 (1.0–1.0)	0.314
Sitting (day)	1.0 (1.0–2.0)	1.0 (1.0–1.0)	2.0 (1.0–2.0)	0.004
Standing (day)	2.0 (2.0–2.0)	2.0 (1.0–2.0)	2.0 (2.0–3.0)	<0.001
ICU Mobility Scale (POD1)	3.0 (1.0–3.0)	3.0 (3.0–4.0)	3.0 (1.0–3.0)	0.004
Numerical Rating Scale (POD2)	3.0 (1.3–5.0)	2.0 (1.0–4.0)	3.0 (2.0–6.0)	0.070
Orthostatic intolerance (POD2), n (%)	30 (43.5%)	9 (25.0%)	21 (63.6%)	0.002
Orthostatic hypotension (POD2), n (%)	34 (49.3%)	15 (41.7%)	19 (57.6%)	0.232
Delirium (≥POD3), n (%)	1 (1.4%)	0 (0.0%)	1 (2.9%)	0.493
Intensive care unit stay (day)	4.0 (4.0–5.0)	4.0 (4.0–4.0)	4.0 (4.0–6.0)	0.013
Hospital stay (day)	23.0 (20.0–29.0)	21.0 (19.0–28.5)	24.0 (22.5–29.0)	0.035
30-day mortality, n (%)	0 (0%)	0 (0%)	0 (0%)	–
90-day mortality, n (%)	0 (0%)	0 (0%)	0 (0%)	–

This study had missing data. Numerical Rating Scale: non-EM group (n = 1); orthostatic intolerance: non-EM group (n = 2); and orthostatic hypotension: non-EM group (n = 2)

EM, early mobilization; POD, postoperative day; ICU, intensive care unit

### Association between EM and barriers to EM

The only factor on POD 2 that significantly differed between the two groups as a barrier to EM was OI, with a higher incidence in the non-EM group (Table 3). Multivariate logistic regression analysis showed that patients with OI were more likely to not achieve EM (≥IMS7) compared with patients without OI (Table 4).

**Table 4.** Association of early mobilization with barriers to early mobilization

Variable	Odds ratio	95% CI	P value
Orthostatic intolerance	7.030	1.750–28.30	0.006
Albumin	0.122	0.012–1.230	0.074
% vital capacity	0.958	0.912–1.010	0.085
Gait speed	0.144	0.008–2.440	0.180
Grip strength	0.901	0.782–1.040	0.150
Blood loss	1.000	0.999–1.010	0.096
Gastric conduit reconstruction	3.080	0.028–345.0	0.640

## Discussion

This study suggests that the incidence of postoperative acute-phase pneumonia is reduced by achieving ambulation by POD 2 in patients undergoing RAMIE for TESCC. In this study, EM was defined using the IMS, and postoperative pneumonia was defined using the ECCG definition and Clavien–Dindo classification. Therefore, it can be inferred that the results of this study are reproducible.

### Association between EM and postoperative pneumonia

Rivas et al.^18)^ previously reported a significant association between postoperative mobilization time and postoperative complications (including pulmonary complications), showing that the incidence of postoperative complications decreased with each hour of increased daily mobilization. However, they also reported that the association between postoperative complications and low levels of mobilization cannot exclude reverse causality as patients with postoperative complications move less^18)^. In this study, the association between EM by POD 2 and postoperative acute-phase pneumonia on POD 3–5 was investigated to clarify temporal relationships. Overall, we found no differences in the risk factors for postoperative pneumonia between the two groups, suggesting that the study was based on internal validity. Therefore, based on our results, we inferred that there is a cause–effect relationship between EM and postoperative pneumonia.

Females were excluded from this study. It has been reported that females have a lower incidence of postoperative pneumonia^30)^ and a higher incidence of OI^31^^,^^32)^ than males. In this study, patients were divided into two groups according to the level of EM, and it is possible that there are more females in the non-EM group due to the influence of OI. If there are more females in the non-EM group, the incidence of pneumonia in the non-EM group may decrease. Therefore, females were excluded from this study because it may be difficult to confirm the association between achieving EM and a lower incidence of postoperative pneumonia.

In abdominal surgery patients, Rivas et al.^18)^ reported that patients with <1.6 hrs/day of mobilization developed pulmonary complications within 48 hrs after surgery, but not those who initiated mobilization with ≥1.6 hrs/day. Haines et al.^15)^ reported that each day of postoperative ≥10 m non-ambulation increased the likelihood of developing a pulmonary complication. Therefore, we infer that the EM group had a lower incidence of postoperative acute-phase pneumonia due to higher mobility by POD 2 and earlier start of ambulation.

### Association between EM and OI

The incidence of OI was higher in the non-EM group than in the EM group. Multivariate logistic regression analysis was adjusted for background factors, and OI was identified as an independent factor limiting EM. Previous studies have shown that OH is not a factor limiting EM^33^^,^^34)^; however, OI has been reported to be associated with limitations of EM^31)^, which was also reported in this study. Therefore, it is more likely that the patients were classified into the EM and non-EM groups based on the difference in the incidence of OI. However, it has also been reported that OI is not directly related to the incidence of postoperative complications^32^^,^^35)^. In this study, it was suggested that OI can have a negative impact on the EM after RAMIE.

### Limitations of this study

This study had several limitations. As most patients with esophageal cancer in Japan are men and have thoracic esophagus and SCC, generalization to many patients who undergo esophagectomy in Japan is possible. However, patients with female sex, cervical and abdominal esophageal cancer, and adenocarcinoma were excluded; therefore, generalization to these patients is not possible. Second, we did not investigate the mechanism by which EM lowers the risk of postoperative pneumonia.

### Implications for future research

This study suggests that patients whose EM was limited to POD 2 because of OI had a higher incidence of postoperative acute-phase pneumonia. The mechanisms and preventive measures of OI are not clear^36)^ and are considered pathological factors that should be investigated in the future to enhance postoperative recovery^37)^. Future studies should, therefore, investigate the association between prolonged mobility time and the incidence of postoperative pneumonia in patients whose EM is limited by OI.

## Conclusion

The introduction of new medical advances, such as RAMIE, has lowered the incidence of postoperative pneumonia in patients following esophagectomy. In this study, EM after RAMIE was suggested to reduce the incidence of postoperative acute-phase pneumonia.

## Acknowledgments

The authors would like to thank the Division of Physical Medicine and Rehabilitation, Okayama University Hospital, and the Graduate School of Health Science Studies, Kibi International University, for their involvement in this study.

The authors would like to thank Editage (www.editage.jp) for English language editing.

## Funding

Not applicable.

## Conflicts of Interest

The authors declare no conflicts of interest.
